# A Novel Image Retrieval Based on Visual Words Integration of SIFT and SURF

**DOI:** 10.1371/journal.pone.0157428

**Published:** 2016-06-17

**Authors:** Nouman Ali, Khalid Bashir Bajwa, Robert Sablatnig, Savvas A. Chatzichristofis, Zeshan Iqbal, Muhammad Rashid, Hafiz Adnan Habib

**Affiliations:** 1 Faculty of Telecommunication and Information Engineering, University of Engineering and Technology, Taxila, Pakistan; 2 Institute of Computer Aided Automation, Computer Vision Lab, Vienna University of Technology, Vienna, Austria; 3 Department of Electrical and Computer Engineering, Democritus University of Thrace, Xanthi, Greece; 4 Department of Computer Engineering, Umm Al Qura University, Makkah, Saudi Arabia; Stanford University Medical Center, UNITED STATES

## Abstract

With the recent evolution of technology, the number of image archives has increased exponentially. In Content-Based Image Retrieval (CBIR), high-level visual information is represented in the form of low-level features. The semantic gap between the low-level features and the high-level image concepts is an open research problem. In this paper, we present a novel visual words integration of Scale Invariant Feature Transform (SIFT) and Speeded-Up Robust Features (SURF). The two local features representations are selected for image retrieval because SIFT is more robust to the change in scale and rotation, while SURF is robust to changes in illumination. The visual words integration of SIFT and SURF adds the robustness of both features to image retrieval. The qualitative and quantitative comparisons conducted on Corel-1000, Corel-1500, Corel-2000, Oliva and Torralba and Ground Truth image benchmarks demonstrate the effectiveness of the proposed visual words integration.

## Introduction

CBIR provides a potential solution to the challenges posed when retrieving images that are similar to the query image [1, 2]. Occlusion, overlapping objects, spatial layout, image resolution, variations in illumination, semantic gap and the exponential growth in multimedia contents make CBIR a challenging research problem [1–3]. In CBIR, an image is represented as a feature vector that consists of low-level image features [2]. The closeness of the feature vector values of a query image to the images placed in an archive determines the output [4].

Color, texture and shape are examples of the global low-level features that can describe the content-based attributes of an image [2]. Color histograms are invariant to changes in scale and rotation [3]. The color features do not represent spatial distribution; moreover the closeness of the color values of two images belonging to different classes results in the output of irrelevant images [1, 2]. Texture features represent spatial variations in the group of pixels and are classified into two categories [5]. Spatial texture techniques are sensitive to noise and distortion, while spectral texture techniques work effectively on square regions by using the Fast Fourier Transform (FFT) [5]. Zhang et al. [6] classify shape features into two categories: region-based and contour-based. Region-based approaches extract shape features from the entire region, and are mostly applied together with color features [6]. Contour-based approaches are applied to extract features from the edges of an image and are sensitive to noise [5].

**Fig 1:**
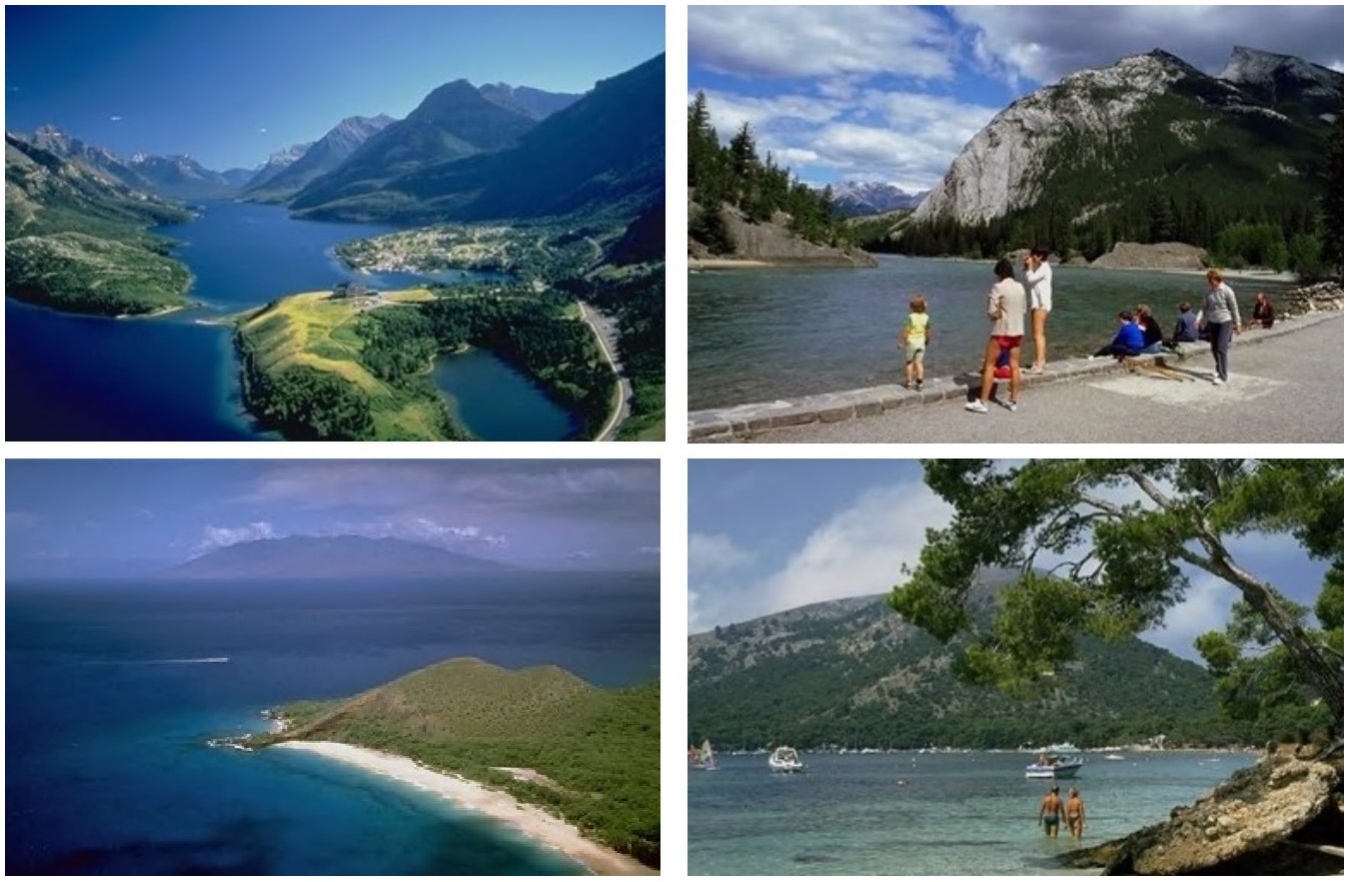
Images of different semantic classes from the Corel image benchmark.

The appearance of a similar view in images belonging to different classes, results in the closeness of the feature vector values; it also decreases the performance of image retrieval [1, 2]. The main focus of the research in CBIR is to retrieve images that are in a semantic relationship with a query image [1, 2]. Fig 1 represents four images of two different classes from the Corel image benchmark with a close visual similarity and semantic likeness. The human eye groups all of these images together as similar in terms of color, while at the same time recognizing a high-level semantic content. In contrast, a closer look leads to the result that the two images in the first row belong to the semantic class Mountains, while the images in the second row belong to the class Beach. While there are visual similarities like sky, clouds, people and water in both of the categories, based on the user preferences during a search, an image retrieval system must be able to retrieve images that meet the specific requirements [1, 2].

In general, CBIR methods can be classified into two groups that employ local and global features [1, 7]. To support the visual queries, i.e. to retrieve visually similar images, mainly global features are used [3]. In most cases, the global features are able to capture an abstract level of semantic similarity [8]. While global features are able to identify the fact that all of the aforementioned images belong to the semantic class “natural landscapes”, usually their results are notoriously noisy [8]. By employing a global feature, a query image of a red tomato on a white background would retrieve a red pie-chart on white paper in the early positions [9]. On the other side of the spectrum, systems that support semantic queries primarily use local features, as they are able to sort the retrieved results more accurately [8–10]. If a user queries an image depicting a mountain, the retrieval system will firstly sort visually similar images that illustrate mountains (a more detailed semantic description). In the sequel, the system will include visually similar images from a higher-level semantic class. According to the recent literature, local features provide slightly better retrieval effectiveness than global features [8, 10, 11].

In recent years, local features such as SIFT [12], Histogram of Oriented Gradients (HOG) [13], SURF [14], Binary Robust Invariant Scalable Keypoints (BRISK) [15] and Maximally Stable Extremal Regions (MSER) [16] have been applied for robust content-based image matching [8, 17, 18]. There are numerous studies on local features that are associated with different applications [8, 10, 17]. Using local features, the representation of the image is mapped into a high-dimensional local feature space. In applications such as Visual Simultaneous Localization And Mapping (VSLAM), panorama construction and object recognition, these extracted features are used directly to find one-to-one matches between depictions [19]. In CBIR, perfect retrieval results have not been reported yet because a single feature-based image representation is not robust for all transformations [20, 21]. The visual features are combined to enhance the effectiveness and reliability of image retrieval [3, 5, 20, 21]. SIFT and SURF are reported as two robust local features [22] and both are evaluated on different image datasets [22–24]. According to the experimental results [23], SIFT is more robust to rotation, change of scale, and is capable of capturing local object edges and shape by using the distribution of the intensity gradients [12]. SIFT performs accurately on the images with a simple background and represents them without noise interference [20, 21]. The performance of SIFT decreases with a complex noisy background and changes in illumination [20, 21, 23]. SURF is reported to be robust to changes in illumination [23] and the SURF descriptor is more distinctive [25]. We show that by integrating the visual words of SIFT and SURF, more precise, effective, and reliable image retrieval results can be obtained.

Keeping these facts in mind, this paper, presents a novel lightweight visual words integration of SIFT and SURF. The local features are extracted from the images; for a compact representation, the feature space is quantized and two codebooks are constructed by using features of SIFT and SURF, respectively. The codebooks consisting of visual words of SIFT and SURF are concatenated and this information is added to the inverted index of the Bag of Features (BoF) [26] representation. The main contributions of this paper are:

1. Image retrieval based on visual words integration of SIFT and SURF.
2. Reduction of the semantic gap between low-level features and high-level image concepts.

## Related Work

Query By Image Content (QBIC) is the first system launched by IBM for image search [1, 3]. After that, a variety of feature extraction techniques are proposed that are based on color, texture, shape and spatial layout [2–5, 27–34]. The visual feature integration is applied to reduce the semantic gap between low-level image features and high-level image concepts [3, 5, 20, 21]. Lin et al. [35] proposed CBIR and applied the low-level feature combination of color and texture. Due to variations of color and texture in the images, a combination of color and texture provides an option to extract the stronger feature [35]. The Color Co-occurrence Matrix (CCM) and the Color Histogram for *K*-Mean (CHKM) is applied to extract the color, while texture is extracted from Difference Between Pixels of Scan Pattern (DBPSP) [35]. The probability of co-occurrence of the same color pixel and an adjacent one is calculated by the use of conventional CCM and is considered as an attribute for that image. The color histogram of two different images with a similar color distribution results in a degradation of the image retrieval performance [2]. Yildizer et al. [36] proposed CBIR for non-texture images and applied Daubechies wavelet transformation to divide an image into high and low frequency bands. The multi-class Support Vector Regression (SVR) model is applied to represent the images in the form of low-level features [36].

To improve the performance of CBIR, Yuan et al. [21] proposed a combination of Local Binary Pattern (LBP) and SIFT. The visual features of SIFT and LBP are extracted separately. Yu et al. [20] proposed the features integration framework of SIFT and HOG with LBP. The weighted average *k*-means clustering is applied to maintain a balance between both features. According to the experimental results [20], the best retrieval performance is obtained by using the features integration of SIFT and LBP. Tian et al. [37] proposed the rotation and scale-invariant Edge Oriented Difference Histogram (EODH). The vector sum and steerable filter are applied to obtain the main orientation of each pixel. A weighted word distribution is obtained by applying the integration of color SIFT and EODH. Karakasis et al. [38] proposed an image retrieval framework by using affine moment invariants as descriptors. The affine moment invariants are extracted with the help of the SURF detector. Wan et al. [39] reported some encouraging results, introducing a deep learning framework for CBIR by training large-scale Convolutional Neural Networks (CNN). According to their conclusions, the features extracted by using a pre-trained CNN model may or may not be better than the traditional hand-crafted features. By applying proper feature refining schemes, the deep learning feature representations consistently outperform conventional hand-crafted features [39].

Lenc et al. [40] combined the descriptors of SIFT and SURF for Automatic Face Recognition (AFR). The framework [40] is based on early features fusion of SIFT and SURF. According to Liu et al. [41], spatial information carries significant information for content verification. The spatial context of local features is represented in binary codes for implicit geometric verification. According to the experimental results [41], the multimode property of local features improves the efficiency of image retrieval. Guo et al. [42] proposed Dot-Diffused Block Truncation Coding (DDBTC), which is based on a compressed data stream, in order to derive image feature descriptors. A DDBTC-based color quantizer and its correspondence bitmap are used to construct the feature space. An image compressed by applying DDBTC provides an efficient image retrieval and classification framework. Liu et al. [43] organized the local features into dozens of groups by applying *k*-means clustering. In this approach, a compact descriptor is selected to describe the visual information of each group. This reorganization of thousands of local features into dozens of groups reduces complexity for a large-scale image search. However, the enhanced retrieval robustness is obtained with a higher computational cost and limited scalability. In this paper, we illustrate how a simple image retrieval approach can provide comparable effectiveness. Based on the experimental results, the proposed approach demonstrates an impressive performance and can be safely recommended as a preferable method for image retrieval tasks. Incorporated into a basic retrieval system that employs the BoF [26] architecture and tested by varying vocabulary sizes, the simple visual words integration of SIFT and SURF outperforms several state-of-the-art image retrieval methods. It is safe to conclude that depending on the image collection, a SIFT and SURF visual words integration framework can yield good retrieval performance with the additional benefits of fast indexing and scalability.

## Proposed Methodology

**Fig 2:**
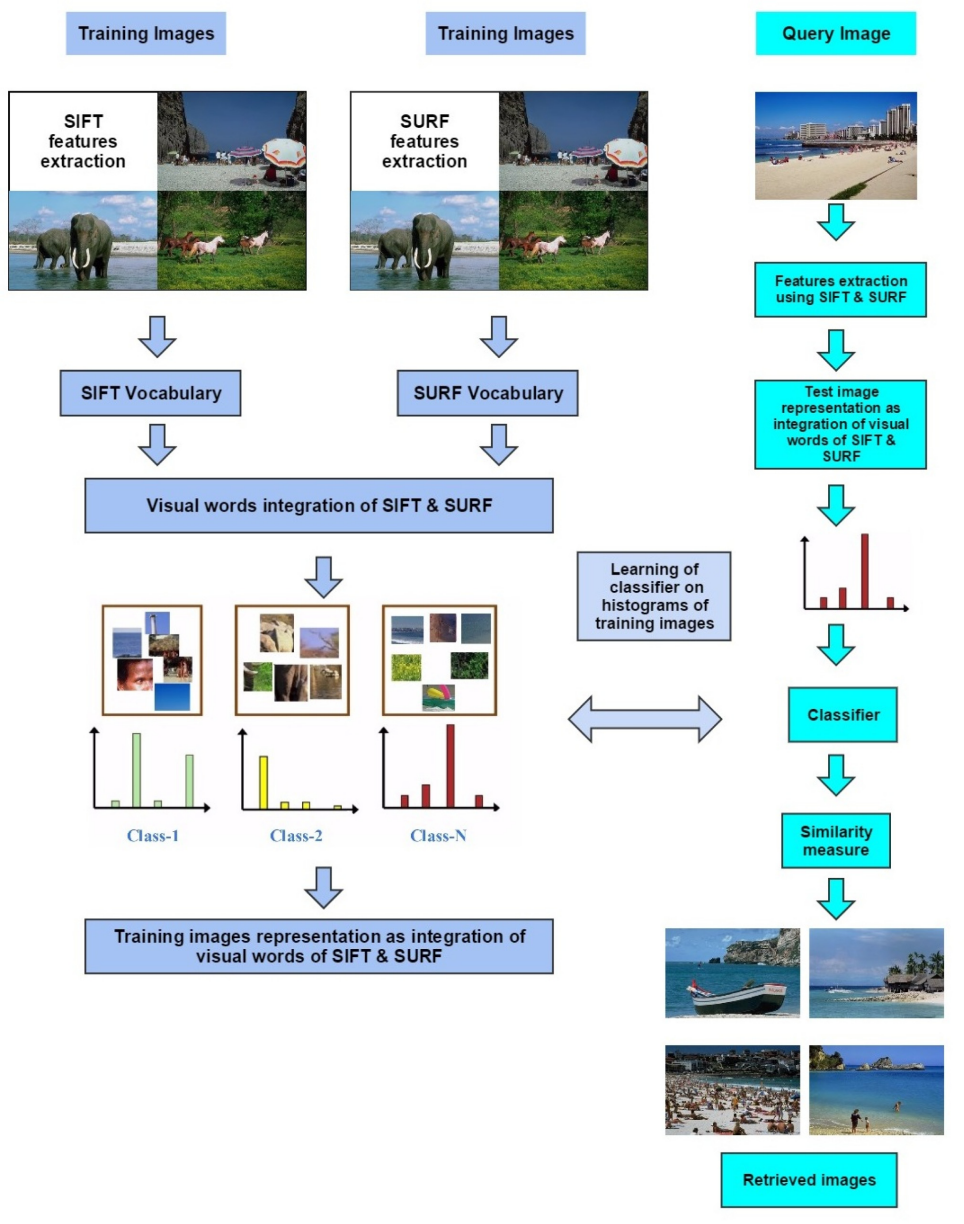
Block diagram of the proposed framework based on the visual words integration of SIFT and SURF.

The proposed image representation is based on the BoF representation [26]. Fig 2 represents the block diagram of the proposed framework. SIFT, SURF, visual words integration using the BoF representation as well as image classification are discussed in detail in the following subsections.

### Scale Invariant Feature Transform (SIFT)

Scale space extrema detection, keypoints localization, orientation assignment and keypoint descriptor are the four major steps for computing the SIFT descriptor [12]. In the first step, the Difference-of-Gaussian (DoG) is applied for the calculation of potential interest points and several Gaussian blurred images are produced by applying different scales to the input image. The DoG is calculated by using the neighborhood blur images. A series of DoG is applied to the scale space and stable keypoints are detected by using the maxima and minima of the Laplacian of Gaussian. In the second step, the extrema are calculated in DoG images for the selection of candidate keypoints. Taylor series is applied to eliminate low contrast and poor localized candidates along the edges. In the third step, the principal orientation is assigned to the keypoints and achieves invariance to image rotation. The fourth step computes the SIFT descriptor across each keypoint. The descriptor gradient orientations and coordinates are rotated relative to the keypoint orientation and provide the orientation invariance. For each keypoint, a set of orientation histograms are created on 4 × 4 pixel neighborhood, with 8 orientation bins in each. This results in feature vectors containing 128 dimensions, SIFT descriptors are invariant to contrast, scale and rotation [12].

### Speeded-Up Robust Features (SURF)

There are two main steps to compute the SURF keypoints and descriptors [14]. The box filter is applied to the integral images for an efficient computation of the Laplacian of Gaussian. Determinants of the Hessian matrix are calculated for the detection of the keypoints. In the second step, every keypoint is assigned to a reproducible orientation by applying the Haar wavelet in the direction of *x* and *y*. A square window is applied around the keypoints and is oriented along the orientations detected before. The Haar wavelets with a size of 2*σ* are calculated by applying the window that is divided into 4 × 4 regular sub-regions and each sub-region contributes values. This results in feature vectors containing 64 dimensions, SURF descriptors are invariant to rotation, change of scale and contrast [14].

### Visual Words Integration of SIFT and SURF

The proposed image representation is based on the visual words integration of SIFT and SURF by using the BoF representation [26]. SIFT and SURF features are extracted from an image. The extracted local features contain visual information about an image. For a compact representation of an image, the feature space is reduced to clusters by applying a quantization algorithm like *k*-means [26]. The cluster centers are called visual words and the combination of visual words represents the visual vocabulary. Two codebooks (visual vocabulary) are constructed by using SIFT and SURF features, respectively. From a given image, SIFT and SURF features are extracted, and then quantized; visual words are assigned to the image by using the Euclidean distance between the visual words and the quantized descriptors. The visual words of SIFT and SURF are concatenated to represent an image in the form of the visual words of SIFT and SURF.

### Image Classification

Support Vector Machines (SVM) are an example of a supervised learning classification method [5]. The kernel method [44] is used in SVM to compute the dot product in the high-dimensional feature space and provides the ability to generate non-linear decision boundaries. The kernel function makes it possible to use the data with no obvious fixed dimensions. The histograms constructed by using the visual words integration of SIFT and SURF are normalized and the SVM Hellinger kernel [45] is applied to the normalized histograms. The SVM Hellinger kernel is selected because of its low computational cost. Instead of computing the kernel values, it explicitly computes the feature map, and the classifier remains linear. The best value for the regularization parameter *C* is determined by using *n*-fold cross validation on the training dataset. The one-against-one [46] approach is applied and for *k* number of classes, *k*. (*k*-1)/2 classifiers are constructed to train the data using two classes.

## Experiments and Results

This section provides the details about the experiments conducted for the evaluation of the proposed framework. The proposed image representation is evaluated on Corel-1000 [47], Corel-1500 [48], Corel-2000 [48], Oliva and Torralba [49], and Ground Truth [50] image benchmarks. SIFT and SURF are used for features extraction, and therefore all of the images are processed in gray scale. Due to unsupervised clustering using *k*-means, all of the experiments are repeated 10 times and average values are reported. For every experiment, training and test datasets are selected randomly. The size of the visual vocabulary is a major parameter that affects the performance of content-based image matching [51, 52]. Increasing the size of the vocabulary increases the performance and a larger vocabulary tends to overfit [51]. Different sizes of visual vocabulary are constructed from a set of training images to find out the best performance of the proposed image representation. The features percentage to construct the visual vocabulary from the training dataset is a major parameter that affects the performance [51]. Different percentages of features per image are used to construct visual vocabulary from the training dataset.

### Weighted Average of SIFT and SURF

The proposed image representation is based on the visual words integration of SIFT and SURF. Differently weighted averages of SIFT and SURF are also calculated to report the second best retrieval performance. The Weighed Average (WA) of SIFT and SURF is calculated by using the following equation:

$$\begin{array}{c} WA=\frac{w*FV_{SIFT}+\left(1-w\right)*FV_{SURF}}{2} \end{array}$$ (1)

where *FV*_*SIFT*_ and *FV*_*SURF*_ are the feature vectors consisting of visual words of SIFT and SURF respectively and 0 < *w* < 1.

### Performance Evaluation

To evaluate the performance of our proposed image representation, we determined the relevant images retrieved in response to a query image. The classifier decision labels determine the class while the classifier decision value (score) is used to retrieve similar images. The Euclidean distance between a query image and the images placed in an archive determines the output of retrieved images. Precision and recall are used to determine the performance of the proposed framework. Precision is used to determine the number of relevant images retrieved in response to the query image and it shows the specificity of the image retrieval system.

$$\begin{array}{c} Precision=\frac{Number\;of\;relevant\;images\;retrieved}{Total\;number\;of\;images\;retrieved} \end{array}$$ (2)

Recall is used to measure the sensitivity of the image retrieval system. Recall is calculated by the ratio of correct images retrieved to the total number of images of that class in the dataset.

$$\begin{array}{c} Recall=\frac{Number\;of\;relevant\;images\;retrieved}{Total\;number\;of\;relevant\;images} \end{array}$$ (3)

### Performance on the Corel-1000 Image Benchmark

**Fig 3:**
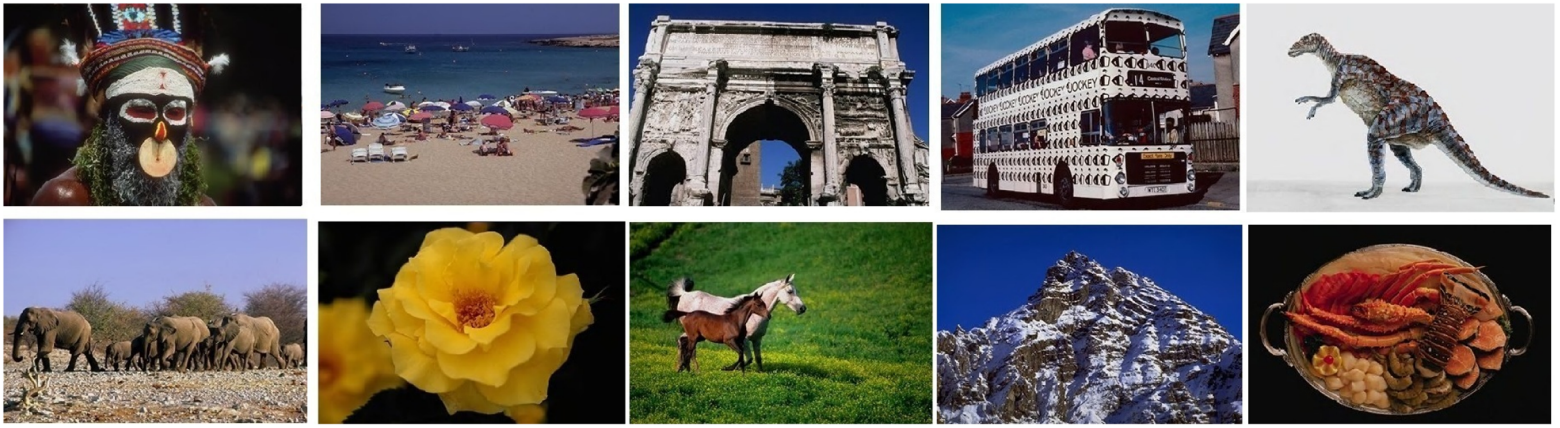
Samples of images from each category of the Corel-1000 image benchmark [47].

The Corel-1000 image benchmark [47] is a sub-set of the Corel image dataset [48] and is extensively used to evaluate CBIR research [20, 37, 53]. The Corel-1000 image benchmark contains 1000 images divided into 10 semantic classes. Fig 3 represents the images from all of the categories from the Corel-1000 image benchmark. The Corel-1000 image benchmark is selected for the evaluation of the proposed image representation and image retrieval precision is compared with existing state-of-the-art CBIR approaches [20, 37, 53]. Testing is performed by a random selection of 500 images from the test dataset. The mean average precision of the proposed image representation is evaluated by using different sizes of vocabulary [50, 100, 200, 400, 600, 800, 1000, 1200]. Different weighted averages of SIFT and SURF are also calculated to find out the second best performance on the Corel-1000 image benchmark. The weighted average values used in the experimental work for SIFT-SURF are 1.0-0.0, 0.9-0.1, 0.8-0.2, 0.7-0.3, 0.6-0.4, 0.5-0.5, 0.4-0.6, 0.3-0.7, 0.2-0.8, 0.1-0.9 and 0.0-1.0, where the first value represents the weight of SIFT and the second value represents the weight of SURF. The best mean average precision is obtained when using the weighted average of 0.7-0.3 (SIFT-SURF). The mean average precision, sigma, and confidence interval (CI) for the top 20 retrievals obtained by using visual words integration and weighted average of 0.7-0.3 (SIFT-SURF) is represented in Tables 1 and 2, respectively.

**Table 1:** Mean average precision for top 20 retrievals (visual words integration).

Vocabulary size & features % used	50	100	200	400	600	800	1000	1200
10%	68.87	71.01	74.05	74.46	74.94	74.54	74.77	74.67
25%	69.62	72.52	74.36	74.75	75.48	75.02	75.2	75.53
50%	68.4	72.13	73.62	75.27	75.55	75.4	75.72	75.4
75%	69.94	71.87	73.24	75.78	74.75	75.53	74.75	74.84
100%	69.87	72.69	74.23	74.04	75.15	75.05	74.62	74.89
**Mean**	69.34	72.04	73.9	74.86	**75.17**	**75.10**	75.01	75.06
**CI**	±0.84	±0.82	±0.57	±0.85	±0.42	±0.47	±0.56	±0.46
**Sigma**	0.68	0.66	0.46	0.68	0.34	0.73	0.45	0.38

**Table 2:** Mean average precision for top 20 retrievals (weighted average 0.7-0.3).

Vocabulary size & features % used	50	100	200	400	600	800	1000	1200
10%	61.95	66.39	67.84	68.89	69.99	70.25	70.38	69.73
25%	62.45	66.86	67.56	67.73	70.2	71.2	70.6	70.21
50%	61.69	67.45	66.55	69.17	69.84	70.39	69.9	69.35
75%	62.03	66.49	67.24	69.84	69.65	70.5	70.15	70.01
100%	62.09	66.37	67.96	68.85	69.95	70.57	70.1	70.2
**Mean**	62.04	66.71	67.43	68.90	69.93	**70.58**	**70.23**	69.90
**CI**	±0.34	±0.57	±0.70	±0.95	±0.25	±0.45	±0.34	±0.45
**Sigma**	0.274	0.457	0.565	0.763	0.202	0.366	0.269	0.363

**Fig 4:**
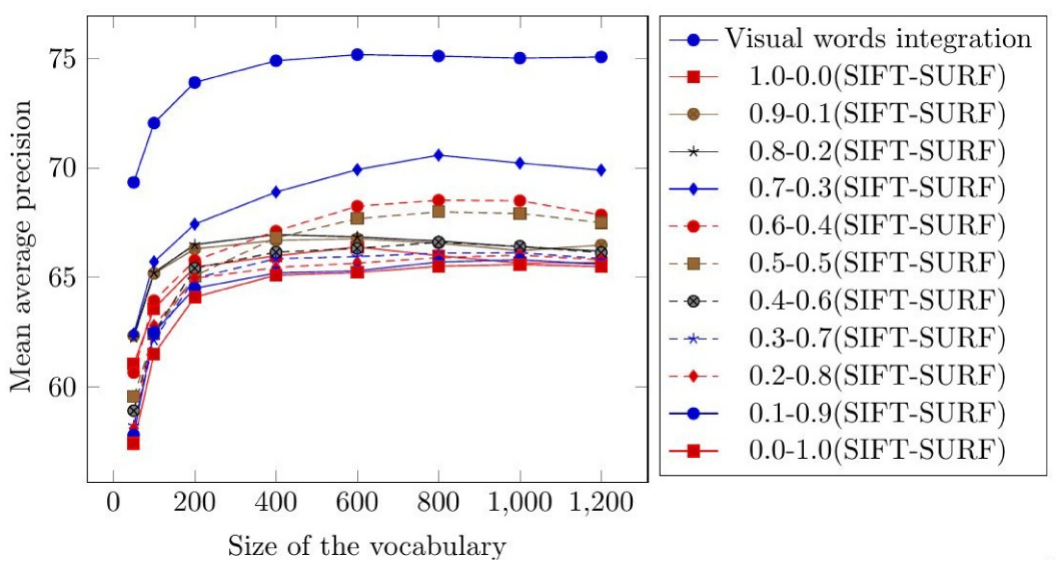
Comparison of mean average precision for top 20 retrievals using the Corel-1000.

According to the experimental results obtained by applying the visual words integration of SIFT and SURF, the best mean average precision of 75.17% is obtained on a vocabulary with a size 600 (by calculating the mean of all columns on the vocabulary of a size of 600 in Table 1). Table 2 represents the mean average precision obtained from the weighted average of 0.7-0.3 (SIFT-SURF). The best mean average precision of 70.58% is obtained on a vocabulary with a size of 800 (by calculating the mean of all columns of the vocabulary of a size of 600 in Table 2). Fig 4 represents the comparison of mean average precision for top 20 retrievals using visual words integration and different weighted averages.

According to the experimental results, the proposed image representation based on the visual words integration of SIFT and SURF significantly enhances the performance of image retrieval. In order to present the sustainable performance of the proposed image representation, we compare the class-wise average retrieval precision for top 20 retrievals with state-of-the-art CBIR approaches [20, 37, 53]. The class-wise comparison of average precision and recall obtained from the proposed framework and state-of-the-art research [20, 37, 53] is presented in Tables 3 and 4, respectively.

**Table 3:** Class-wise comparison of precision for top 20 retrievals.

Class and Method	Visual words integration SIFT-SURF	Weighted average (0.7-0.3) SIFT-SURF	Color SIFT- EODH [37]	Spatial BoF [53]	SIFT-LBP [20]	HOG-LBP [20]
Africa	60.08 ± 1.94	52.68 ± 0.82	**74.6**	**64**	57	55
Beach	**60.39** ± 1.39	56.32 ± 1.70	37.8	54	**58**	47
Buildings	**69.66** ± 3.74	**69.03** ± 1.54	53.9	53	43	56
Buses	93.65 ± 0.84	86.35 ± 1.52	**96.7**	**94**	93	91
Dinosaurs	**99.88** ± 0.054	**99.68** ± 0.16	99	98	98	94
Elephants	**70.76** ± 1.90	67.55 ± 1.58	65.9	**78**	58	49
Flowers	**88.37** ± 0.76	85.99 ± 0.67	**91.2**	71	83	85
Horses	82.77 ± 0.70	76.37 ± 0.97	**86.9**	**93**	68	52
Mountains	**61.08** ± 0.71	**58.85** ± 1.03	58.5	42	46	37
Food	**65.09** ± 1.76	53.00 ± 1.59	**62.2**	50	53	55
**Mean**	**75.17**	70.58	**72.67**	69.7	65.7	62.1

**Table 4:** Class-wise comparison of recall for top 20 retrievals.

Class and Method	Visual words integration SIFT-SURF	Weighted average (0.7-0.3) SIFT-SURF	Color SIFT- EODH [37]	Spatial BoF [53]	SIFT-LBP [20]	HOG-LBP [20]
Africa	12.02 ± 0.39	10.68 ± 0.16	**14.92**	**12.80**	11.40	11.00
Beach	**12.08** ± 0.28	11.34 ± 0.34	7.56	10.80	**11.60**	9.40
Buildings	**13.93** ± 0.75	**13.53** ± 0.31	10.78	10.60	8.60	11.20
Buses	18.73 ± 0.17	17.30 ± 0.30	**19.34**	**18.80**	18.60	18.20
Dinosaurs	**19.98** ± 0.01	**19.95** ± 0.03	19.80	19.60	19.60	18.80
Elephants	**14.15** ± 0.38	13.52 ± 0.32	13.18	**15.60**	11.60	9.80
Flowers	**17.67** ± 0.15	17.30 ± 0.13	**18.24**	14.20	16.60	17.00
Horses	16.55 ± 0.14	15.11 ± 0.19	**17.38**	**18.60**	13.60	10.40
Mountains	**12.22** ± 0.14	**11.78** ± 0.21	11.70	8.40	9.20	7.40
Food	**13.02** ± 0.35	10.65 ± 0.32	**12.44**	10.00	10.60	11.00
**Mean**	**15.03**	14.12	**14.53**	13.94	13.14	12.42

**Fig 5:**
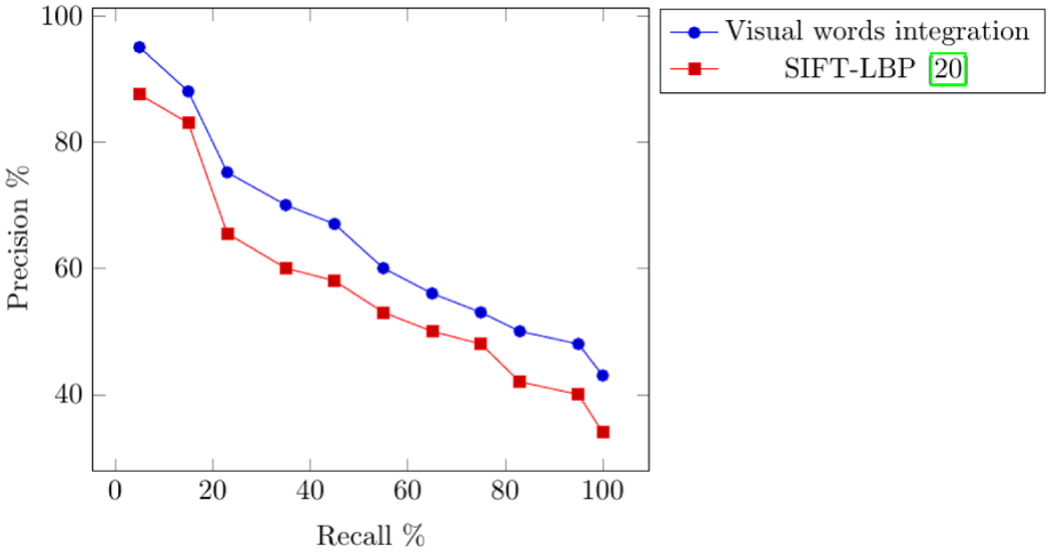
Precision-recall curve obtained using the Corel-1000 image benchmark.

**Fig 6:**
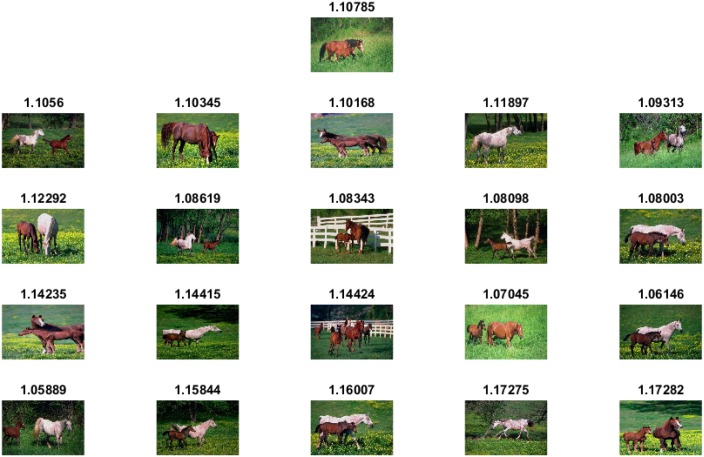
Image retrieval results for the class Horses.

**Fig 7:**
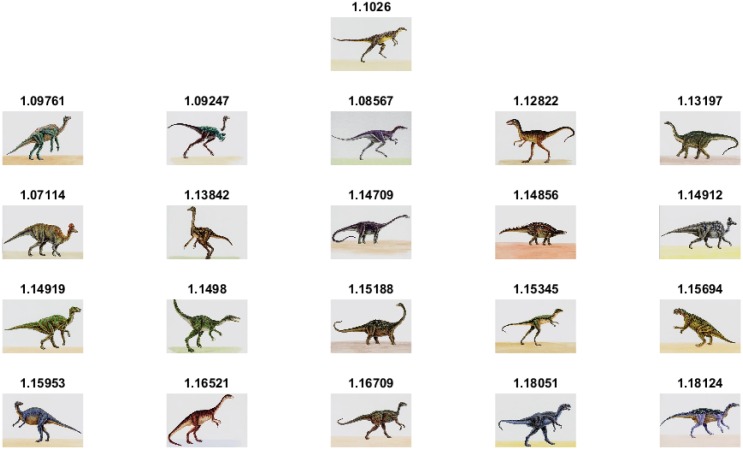
Image retrieval results for the class Dinosaurs.

**Fig 8:**
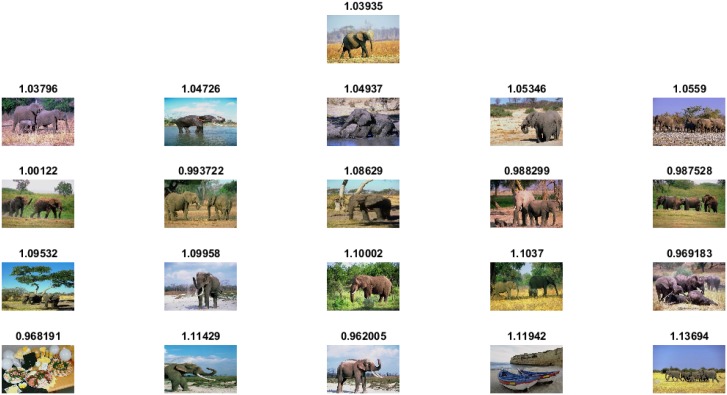
Image retrieval results for the class Elephants.

**Fig 9:**
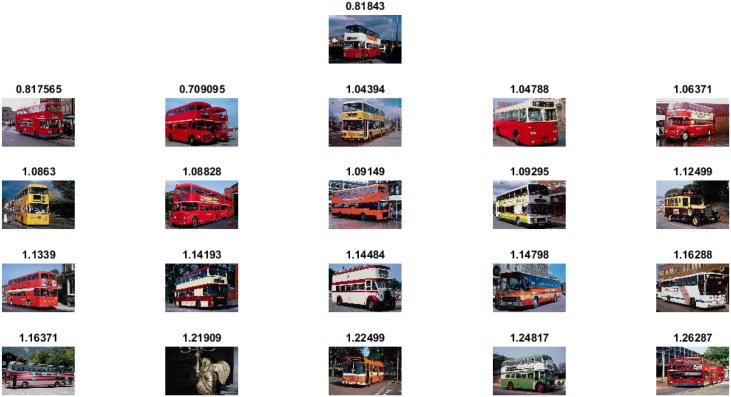
Image retrieval results for the class Buses.

The experimental results and comparisons conducted using the Corel-1000 image benchmark prove the robustness of the proposed image representation based on the visual words integration of SIFT and SURF. The mean average precision value obtained from the proposed framework is higher than that of the the existing state-of-the-art research [20, 37, 53]. Fig 5 represents precision-recall curve obtained using Corel-1000 image benchmark. The image retrieval results obtained from the proposed framework are represented in Figs 6–9. The single image displayed in the first row is the query image, and the numerical value displayed at the top of each image is the classifier decision value (score) of the respective image.

### Performance on the Corel-1500 Image Benchmark

**Fig 10:**
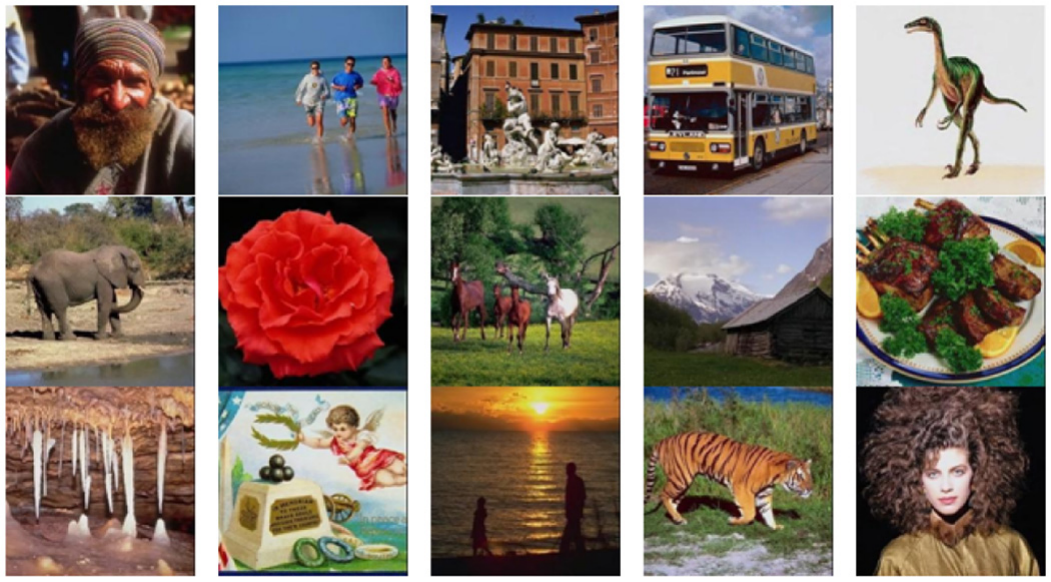
Samples of images from each category of the Corel-1500 image benchmark [48].

**Fig 11:**
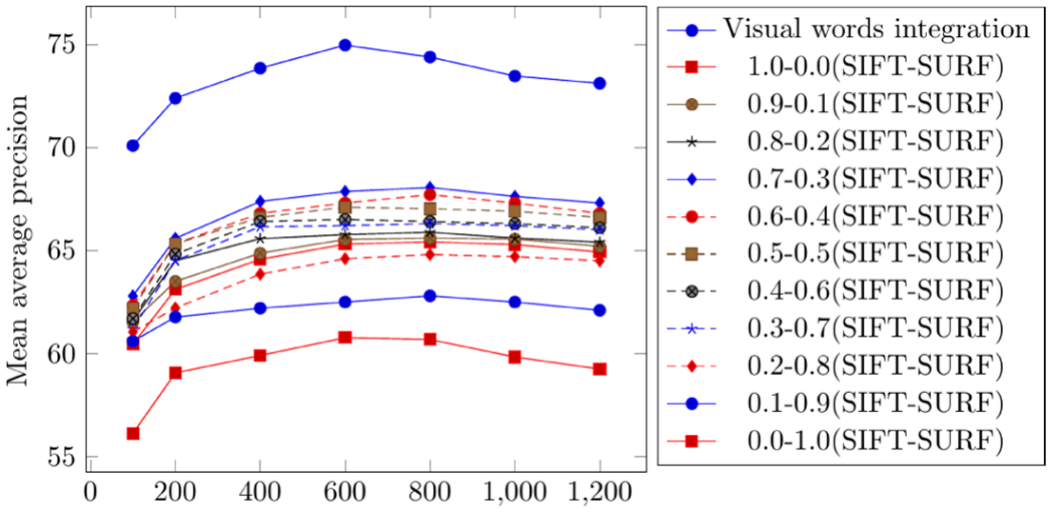
Comparison of mean average precision using the Corel-1500 image benchmark.

The Corel-1500 image benchmark contains 1500 images (divided into 15 semantic classes) and is a sub-set of the Corel image dataset [48]. Fig 10 represents the images from all of the categories from the Corel-1500 image benchmark. Testing is performed by a random selection of 750 images from the test dataset. Fig 11 represents the comparison of mean average precision using visual words integration and different weighted averages.

According to the experimental results, the best mean average precision obtained from the visual words integration of SIFT and SURF on a vocabulary with a size of 600 is 74.95%. The best mean average precision obtained using the weighted average of 0.7-0.3 (SIFT-SURF) on a vocabulary with a size of 800 is 68.05%. The visual words integration of SIFT and SURF significantly enhances the performance of image retrieval. The comparison of precision and recall obtained from the proposed framework and state-of-the-art research [54] is presented in Table 5.

**Table 5:** Comparison of precision and recall using the Corel-1500 image benchmark.

Performance/Method	Visual words integration SIFT-SURF	Weighted average (0.7-0.3) SIFT-SURF	SQ + Spatiogram [54]	GMM + mSpatiogram [54]
Precision	**74.95** ± 1.60	68.05 ± 1.92	63.95	74.10
Recall	**14.99** ± 0.32	13.15 ± 0.38	12.79	13.80

### Performance on the Corel-2000 Image Benchmark

**Fig 12:**
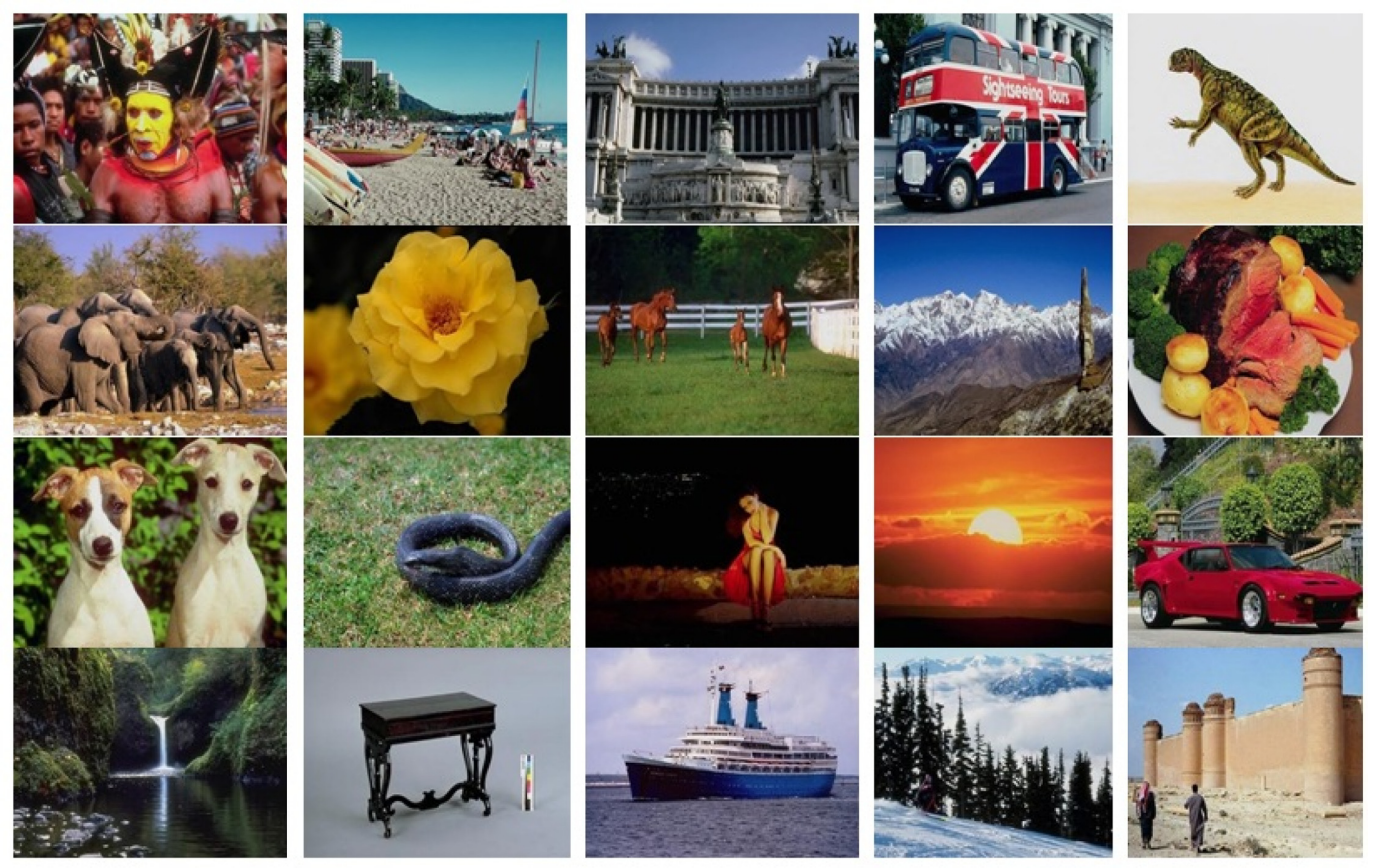
Samples of images from each category of the Corel-2000 image benchmark [48].

**Fig 13:**
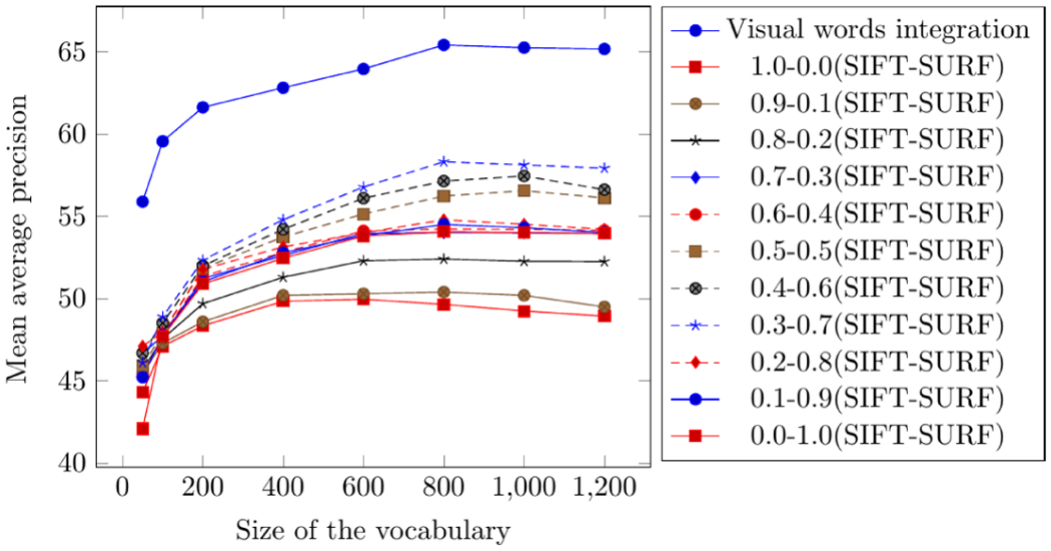
Comparison of mean average precision using the Corel-2000 image benchmark.

The Corel-2000 image benchmark contains 2000 images (divided into 20 semantic classes) and is a sub-set of Corel image dataset [48]. Fig 12 represents the images from all of the categories from the Corel-2000 image benchmark. Testing is performed by a random selection of 600 images from the test dataset. Fig 13 represents the comparison of mean average precision using visual words integration and different weighted averages.

According to the experimental results, the best mean average precision obtained from the visual words integration of SIFT and SURF on a vocabulary with a size of 800 is 65.41%. The best mean average precision of 58.31% is obtained when using the weighted average of 0.3-0.7 (SIFT-SURF). The visual words integration of SIFT and SURF significantly enhances the performance of image retrieval. The comparison of the mean average precision obtained from the proposed frame work and state-of-the-art research [55, 56] is presented in Table 6.

**Table 6:** Comparison of mean average precision using the Corel-2000 image benchmark.

Performance/Method	Visual words integration SIFT-SURF	MissSVM [55]	MI-SVM [56]
**Mean**	**65.41** ± 0.99	65.2	54.6

### Performance on the Oliva and Torralba (OT-Scene) Image Benchmark

**Fig 14:**
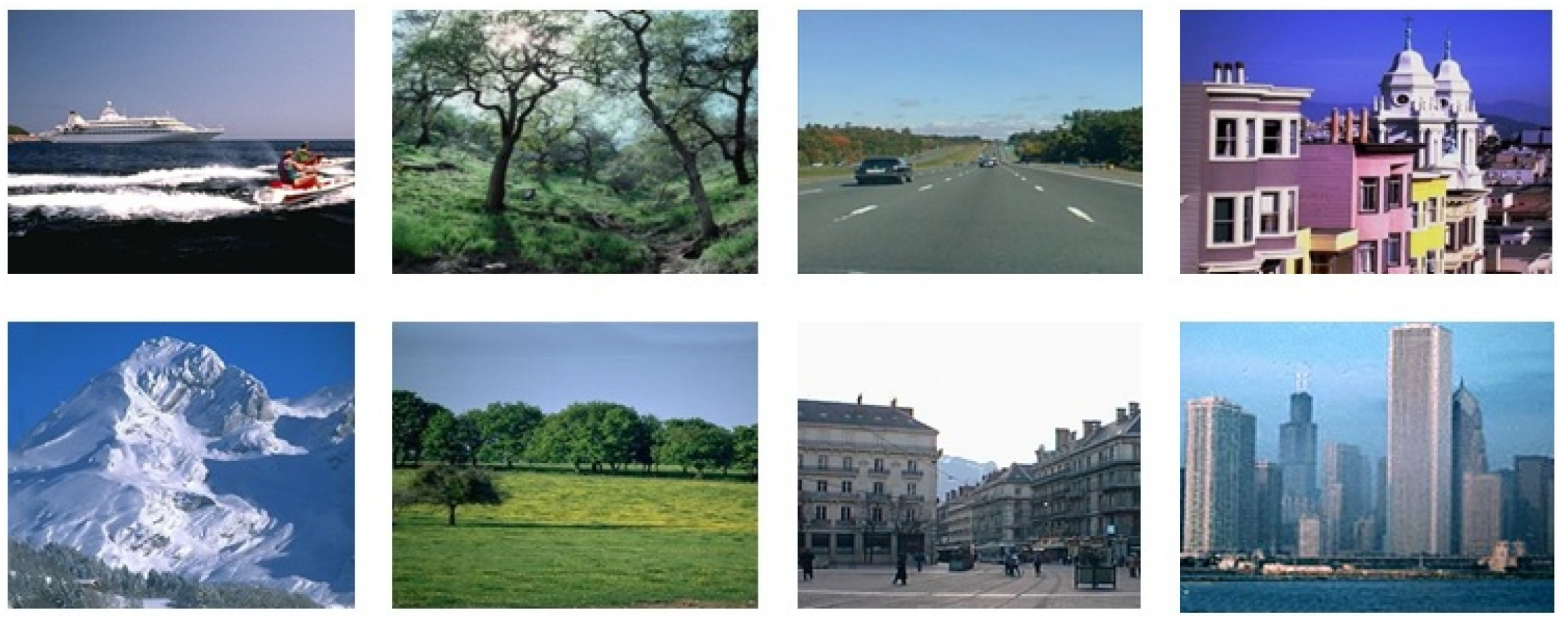
Samples of images from each category of the OT-Scene image benchmark [49].

**Fig 15:**
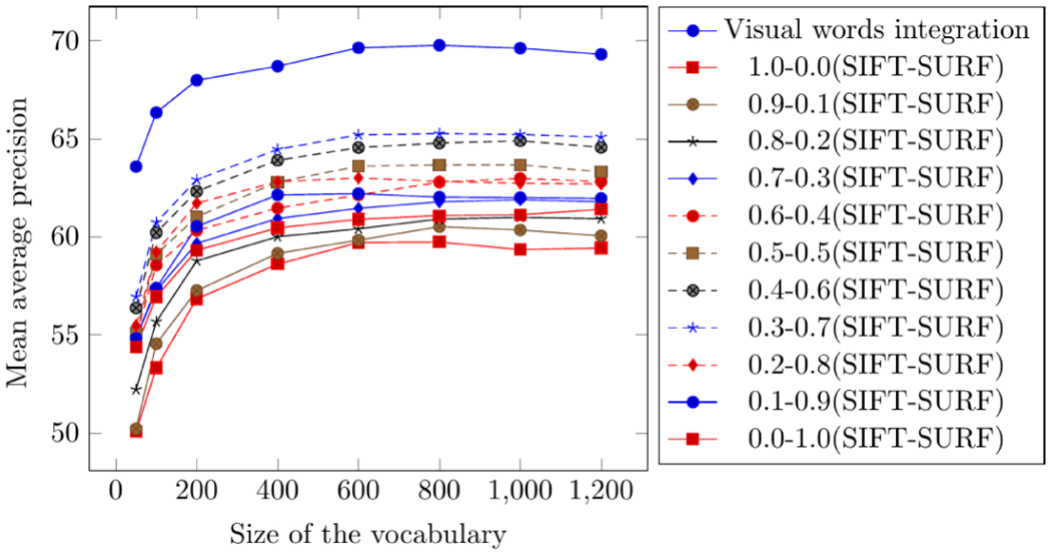
Comparison of mean average precision using the OT-Scene image benchmark.

The Oliva and Torralba (OT-Scene) image benchmark was created by MIT and there are 2688 images that are divided into 08 classes. Fig 14 represents the images from all of the categories from the OT-Scene image benchmark. Testing is performed by a random selection of 600 images from the test dataset. Fig 15 represents the comparison of mean average precision using visual words integration and different weighted averages. The comparison of the mean average precision obtained from the proposed frame work and state-of-the-art CBIR research [57, 58] is presented in Table 7.

**Table 7:** Comparison of mean average precision using the OT-Scene image benchmark.

Performance/Method	Visual words integration SIFT-SURF	Weighted average (0.3-0.7) SIFT-SURF	Feature extraction with morphological operators [57]	Min Max Fusion [58]
**Mean**	**69.75** ± 0.40	65.25 ± 0.52	60.7	51.04

According to the experimental results, the best mean average precision obtained using visual words integration and weighted average of 0.3-0.7 (SIFT-SURF) is 69.75% and 65.25%, respectively. The visual words integration of SIFT and SURF significantly enhances the performance of image retrieval.

### Performance on the Ground Truth Image Benchmark

**Fig 16:**
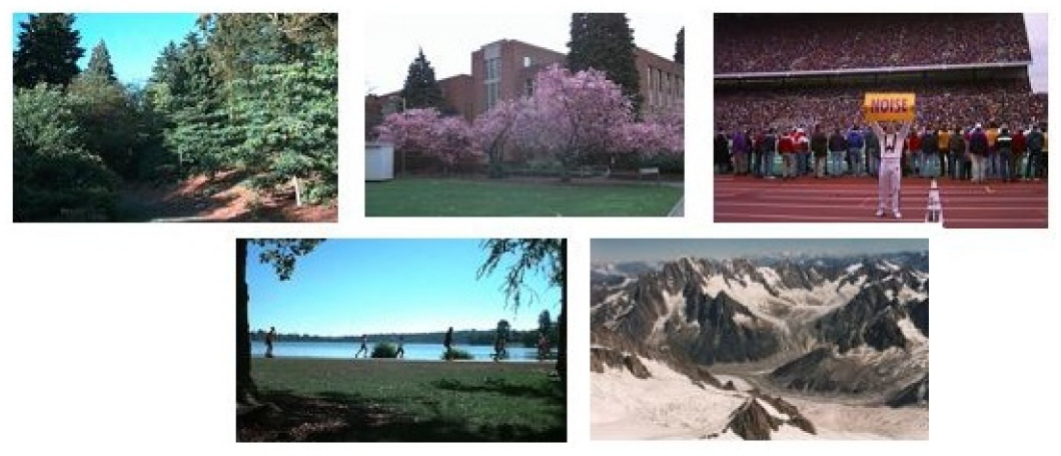
Samples of images from 05 classes of the Ground Truth image benchmark [50].

Ground Truth image benchmark [50] was created by University of Washington and has been previously used for the evaluation of CBIR research [36, 59, 60]. There are a total of 1109 images that are divided into 22 semantic classes. In order to perform a clear comparison with existing state-of-the-art CBIR research [36, 59, 60], we selected 228 images from 05 different classes (Arbor Greens, Cherries, Football, Green Lake and Swiss Mountains), shown in Fig 16. Different sizes of the visual vocabulary are constructed from the training dataset [10, 20, 50, 75, 100] to sort out the best performance of the proposed framework. The best mean average precision is obtained on a vocabulary with a size of 75 with a value of 83.53%. The comparison of the mean average precision obtained from the proposed framework and existing state-of-the-art research [36, 59, 60] is presented in Table 8.

**Table 8:** Comparison of mean average precision using Ground truth image benchmark.

Performance/Method	Visual words integration SIFT-SURF	SVM ensembles [59]	Wavelet based CBIR [60]	SVR ensembles [36]
**Mean**	**83.53** ± 1.50	81.33	62.80	59.09

Experimental results and comparisons conducted on the Ground truth image benchmark prove the robustness of proposed framework based on the visual words integration of SIFT and SURF. The mean average precision obtained from the proposed visual words integration is higher than that of the existing state-of-the-art research [36, 59, 60].

## Conclusion and Future Directions

The semantic gap between low-level visual features and high-level image concepts is a challenging research problem of CBIR. SIFT and SURF are reported as two robust local features and the integration of visual words of SIFT and SURF adds the robustness of both features to image retrieval. As shown by the experimental results, the proposed image representation demonstrates an impressive performance and can be safely recommended as a preferable method for image retrieval tasks. It is safe to conclude that depending on the image collection, the visual words integration of SIFT and SURF can yield good retrieval performance with the additional benefits of fast indexing and scalability. In future, we plan to evaluate our framework for large scale image retrieval (ImageNet or Flicker) by replacing SVM with state-of-the-art classification technique such as deep learning.
